# Effects of a polyphenol‐rich proprietary blend of freeze‐dried fruits and vegetables on serum proteome, cognitive function, and health in older adults at risk of cognitive and functional decline

**DOI:** 10.1002/alz70860_105264

**Published:** 2025-12-23

**Authors:** Mary O'Leary, Joanna Bowtell, Megan Richards, Abbie Palmer, Adam Bloomfield, Esra Bozbas, Celeste Lugtmeijer, George Vere, Raif Yuecel, Ana Rodriguez‐Mateos, Zicheng Zhang, Jonathan Tang, Clive G Ballard, Anne Corbett

**Affiliations:** ^1^ University of Exeter, Exeter, Exeter, United Kingdom; ^2^ University of Exeter, Exeter, United Kingdom; ^3^ King's College London, London, United Kingdom; ^4^ Norwich Medical School, Norwich, United Kingdom

## Abstract

**Background:**

The Mediterranean diet (MD) is associated with cardiovascular and cognitive health benefits. However, it is challenging for Western populations to replicate the recommended blend and quantity of fruits and vegetables. Polyphenols are a key bioactive found in MD with antioxidant and anti‐inflammatory properties, and may also influence extracellular vesicle (EV) dynamics. DailyColors™, a polyphenol‐rich formulation inspired by the MD, incorporates extracts from 16 fruits, vegetables, and herbs. This study aimed to evaluate the cognitive and physiological effects of 60 days of supplementation with DailyColors™ in overweight older adults in the PROTECT‐UK cohort.

**Methods:**

50 UK adults aged 50 years and older with a BMI ≥ 25 were recruited for a randomised, double‐blind, placebo‐controlled study conducted to evaluate cognitive and physical fitness via the PROTECT‐UK online platform. Participants were randomly assigned to receive either a medium dose (750 mg, ∼300 mg polyphenols), a high dose (2000 mg, ∼750 mg polyphenols) of DailyColors™, or a placebo for 60 days. Additionally, a subset of 45 participants (*n* = 15 per group) underwent further assessments, including blood pressure monitoring, phenolic metabolite analysis, evaluation of circulating EVs, and tandem‐mass‐tagged serum proteomics in blood.

**Result:**

Sixty days of DailyColors™ supplementation improved cognitive function, with significant improvements in attentional reaction time and accuracy in the intervention groups. Enhanced physical fitness was observed in the Timed Stand Test in the high‐dose group and in the Chair Stand Test across all groups. Proteomic analysis showed that both medium and high doses of DailyColors™ decreased protein expression in pathways associated with inflammation and immunity, vesicle‐mediated transport. Expression of proteins known to inhibit high density lipoprotein (HDL) assembly was also diminished. No such effects were observed in the placebo group. No significant effects were observed on the size and concentration of total EVs.

**Conclusion:**

DailyColors™ supplementation may improve cognitive function, physical fitness, and overall health in older, overweight individuals, possibly replicating the anti‐inflammatory benefits of the MD. This trial provides proof of concept for rapid, efficient delivery of hybrid trials through the PROTECT platform.

